# The Study of Nutrient Intake and Adolescent Girls’ Quality of Life in a Rural Area of Indonesia

**DOI:** 10.3390/children9081248

**Published:** 2022-08-19

**Authors:** Puspa Sari, Dewi Marhaeni Diah Herawati, Meita Dhamayanti, Dany Hilmanto

**Affiliations:** 1 Doctoral Study Program, Faculty of Medicine, Universitas Padjadjaran, Bandung 45363, Indonesia; 2 Department of Public Health, Faculty of Medicine, Universitas Padjadjaran, Bandung 45363, Indonesia; 3 Department of Child Health, Hasan Sadikin Hospital, Faculty of Medicine, Universitas Padjadjaran, Bandung 45363, Indonesia

**Keywords:** nutrient, adolescent girls, quality of life

## Abstract

An inadequate nutrient intake correlates with malnutrition, a problem affecting many adolescents worldwide. Nutrient intake is associated with quality of life (QoL). Our study analyzed the relationship between nutrient intake and adolescents’ QoL. We conducted a cross-sectional study. Through simple random sampling, 157 adolescent girls were selected. Nutrition status was assessed using anthropometric measurements. Nutrient intake was collected using the food frequency questionnaire (FFQ). WHOQOL BREF was used to explore adolescent girls’ quality of life. The median of nutrient intake: energy (908.25 kcal); protein (24.16 g); carbohydrate (128.89 g); fat (21.89 g); vitamin A (77.10 mg); vitamin E (1.40 mg); vitamin B1 (0.19 mg); vitamin B2 (0.29 mg); vitamin B6 (0.45 mg); folic acid (35.13 mg); vitamin C (12.60 mg); calcium (197.46 mg); magnesium (93.72 mg); iron (2.64 mg); and zinc (2.09 mg). The adolescents’ QoL scores were physical health 44 (25–81), psychological domain 56 (19–94), social relationships 56 (19–94), and environmental domain 56 (31–100). The strongest correlations were between (1) physical health with carbohydrates, vitamin C, and fat; (2) psychological domain with calcium; (3) social relationships with carbohydrates and vitamin C; and (4) environmental domain with BMI and zinc. There was a significant positive correlation between the intake of some nutrients and adolescents’ QoL, despite the observation of some significant negative correlations. The findings of this study indicate that more attention should be focused on adolescents’ nutrient intake in order to improve their QoL.

## 1. Introduction

According to the World Health Organization (WHO), adolescence is defined as youth aged 10–19 years [1]. There are more than 500 million adolescent girls living in low- and middle-income countries [2]. The number of adolescents in Indonesia, based on the Central Statistics Agency in 2018, was 63.82 million [3]. With that number, the health and welfare of adolescents must be prioritized. In order to ensure the health and well-being of the increasing number of adolescents, the country at all levels must ensure that adolescents are able to grow and develop appropriately in an environment free from various health problems [4].

A large number of adolescents in Southeast Asia suffer from malnutrition [4,5]. As inadequate nutrition causes create chronic lifetime health problems, an assessment of adolescents’ nutritional status is critical. According to Indonesia Baseline Health Research, in 2018, as many as 96.8% of Indonesia’s adolescents aged 10–14 years and 96.4% of adolescents aged 15–19 years did not consume vegetables or fruit, which, as is known, are foods rich in essential micronutrients [6]. As protein is contained in vegetables, a lack of vegetable consumption can cause protein deficiency. The total nutrient intake and energy needs of adolescents during puberty exceed those at any other stage of life as a result of increasing skeletal mass, body fat, and lean body mass. Adequate nutrition is the foundation for a healthy lifestyle and appropriate development. One of the United Nation’s Sustainable Development Goals is to address the nutritional needs of adolescent girls [1].

Nutrient intake correlates to life domains, including social interactions, personal satisfaction, economics, and psychological well-being, which influence the quality of life (QoL) [7]. QoL is a concept that varies between children, adolescents, and adults. According to the WHO, QoL is an individual’s perception of one’s position in everyday life, according to the culture and the value system, in which they live and in relation to their goals, hopes, desires, concerns [8]. QoL involves an individual’s mental, physical, and social well-being [9,10,11,12]. From an adolescent perspective, QoL relates to the transitions between normal and expected stages of development. Therefore, adolescents’ QoL is threatened by factors that impede development [13], and complete nutrition is at the foundation of normal development [14]. There are several generic measures of QoL that are widely used worldwide, including the WHOQOL-BREF (World Health Organization Quality of Life Assessment), which was developed by the WHO. This assessment measures four domains of QoL: physical, psychological, social, and environmental, with its 26 items [15]. The explanation for each domain was: (1) Domain 1: Physical health which can affect an individual’s activities. Physical health includes activities of daily living, medical needs and medical assistance, energy and fatigue, mobility, pain and discomfort, sleep and rest, and work capacity; (2) Domain 2: Psychological means psychological aspect, related to the mind of the individual state. Mental refers to the adaptation ability to various developmental demands according to their abilities; (3) Domain 3: Social domain refers to aspects of social relations. The relationship between two or more individuals where individual behavior will influence each other. Social relationships include personal relationships, social support, and sexual activity; (4) Domain 4: Environment refers to the environmental aspect, such as the individual’s place of residence, the availability of a place to live to do all activities in life, including advice and infrastructure that can support life [16].

There are a limited number of studies regarding the nutrient intake and QoL of adolescents. Some studies have focused only on either the nutritional status or the QoL of adolescents in Indonesia, especially in rural areas. Hence, this study focused on the relationship between adolescents’ nutrient intake and their QoL and aimed to determine the most important factors that influence adolescents’ QoL.

## 2. Materials and Methods

### 2.1. Study Design and Sampling

A cross-sectional analytical study was conducted involving 157 adolescent girls selected by simple random sampling in the Soreang subdistrict of Bandung, Indonesia from October to December 2020. Adolescent girls (15–19 years) who were healthy, nonpregnant, and nonlactating during the study period were included in the study. In addition, subjects who were sick were excluded. The consideration of choosing adolescent girls was as the preparation for healthy women after they graduate from high school. Moreover, the majority of adolescent girls in rural areas will get married after the end of high school. Adolescent girls will become mothers. Moreover, paying attention to food intake is crucial.

The sample size was calculated by G*Power 3.1.9.7 with exact, correlation statistical test, sample size at 5% level of significance, and 95% power; the minimal sample was 138. Data collection was carried out during the COVID-19 pandemic with health protocols, but participants who were willing to participate in small numbers met the minimum sample criteria.

### 2.2. Data Collection

The demographic variables included the participants’ age in years, parents’ education and occupations, living conditions, number of siblings, and body mass index (BMI). In Indonesia, all citizens must undertake twelve years of compulsory education, which consists of six years at elementary level and at secondary levels. Moreover, we analyzed differences in parents’ education levels on adolescent quality of life. Macronutrient intake was analyzed, energy, protein, fat, and carbohydrates. Data on energy, and macronutrient intake were reported as kcal per day and as grams per day, respectively. The essential micronutrients such as vitamin A (mg), vitamin E (mg), vitamin B1 (mg), vitamin B2 (mg), vitamin B6 (mg), folic acid (mg), vitamin C (mg), calcium (mg), magnesium (mg), iron (mg), and zinc (mg) were also calculated as mg per day. This study analyzed adolescents’ QoL from four domains: physical activity, psychological domain, social relationships, and environmental domain.

#### 2.2.1. Anthropometry Measurement

Nutrition status was assessed using anthropometric measurements. In this study, nutritional status and nutrient intake were considered to be independent variables. BMI was analyzed from anthropometric measurements, weight was measured to the nearest 0.5 kg on a mass body scale, and height was measured to the nearest 0.1 cm using a stadiometer. According to the WHO, nutritional status was divided into five categories, including underweight (below 18.5), normal (18.5–24.9), pre-obesity (25.0–29.9), obesity class 1 (30.0–34.9), and obesity class 2 (35.0–39.9) [17].

#### 2.2.2. Food Frequency Questionnaire (FFQ)

Normal nutrient intake was collected using the food frequency questionnaire (FFQ), categorized into two groups, including macronutrients and micronutrients. We analyzed energy (kcal), protein (g), carbohydrate (g), and fat (g) as macronutrient intakes, and vit A (mg), vit E (mg), vit B1 (mg), vit B2 (mg), vit B6 (mg), folic acid (mg), vit C (mg), calcium (mg), magnesium (mg), iron (mg), and zinc (mg) as micronutrient intakes. We used the nutrient composition of Indonesia reference as a standard for nutrient intake [18]. Nutri Survey software was used to analyze nutrient intake. Nutri Survey is the English translation of a professional German nutrition software (EBIS-pro). It contains all valuable functions which are typical for this kind of software (nutrient analysis and calculation of energy requirements, planning of diets, diet history, and food frequency) [19].

#### 2.2.3. Quality of Life Measurement

There are various assessments of the QoL among children and adolescents of which the WHOQOL-BREF is one. The WHO QoL-BREF was used to determine the QoL of the study population. The WHO worked with 15 regions worldwide to develop two instruments for measuring the QoL (WHOQOL-100 and WHOQOL-BREF), which can be used in different cultures; this instrument has many uses [20]. The WHO quality of life questionnaire used has been translated into Indonesian [21]. This instrument categorizes QoL into four domains. Each domain value is scaled in a positive direction (higher scores indicate higher QoL scores). The average score of the items in each domain is used to calculate the value of the domain. The score is multiplied by four to make the domain value comparable to the value used in the WHOQOL-100. The first transformation method changes the score to a range of 4–20, comparable to the WHOQOL-100. The second transformation method converts the domain values to a scale of 0–100. The WHOQOL-BREF instrument was used to measure adolescents’ QoL. This tool assesses the QoL based on several domains, namely (1) physical, (2) psychological, (3) social, and (4) environmental domains [22,23,24].

### 2.3. Data Analysis

All statistical analyses were performed using IBM SPSS Version 27.0 for Windows. Descriptive statistics were used in order to understand the distribution. The Kolmogorov–Smirnov test was applied to test the normality of the data. Among the variables that were not normally distributed, the median (interquartile) was applied. The nutrient intake and adolescents’ QoL were assessed to analyze the correlation between both variables. Associations were tested using the Somers ’D test to assess the association between nutrient intake and adolescents’ QoL. The correlation scores were divided into (1) 0 < r < 0.3 (weak positive correlation), (2) 0.3 < r < 0.7 (moderate positive correlation), (3) r > 0.7 (strong positive correlation), (4) −0.5 < r < −0.3 (moderate negative correlation), and for −0.3 < r < −0.1 (weak negative correlation) [25]. The dependent variable was numerical; therefore, a multivariate linear statistical model for regression was done. *P-values less than 5% (0.05) mean the statistical significance of the dependent variable with the independent variables.*

## 3. Results

### 3.1. Participants’ Characteristic

A total of 157 participants in this study were adolescents aged 15–19 years (Table 1). The level of parent education is mostly at the level of elementary. Trading is the most common occupation of participants’ fathers, with a mother as a house wife. Most adolescents lived with family, with two or more siblings. Regarding nutritional status (Table 1), the participants were normal weight, and only a few participants were obesity-1 and obesity-2. Surprisingly, malnutrition still occurred among adolescent girls in the Soreang subdistrict.

### 3.2. Nutrient Intake and Quality of Life

Table 2 shows the median of the participants’ nutrient intake. The median of all nutrient intake was energy (908.25 kcal); protein (24.16 g); carbohydrate (128.89 g); fat (21.89 g); vitamin A (77.10 mg); vitamin E (1.40 mg); vitamin B1 (0.19 mg); vitamin B2 (0.29 mg); vitamin B6 (0.45 mg); folic acid (35.13 mg); vitamin C (12.60 mg); calcium (197.46 mg); magnesium (93.72 mg); iron (2.64 mg); and zinc (2.09 mg). On the other hand, the median of the participants’ QoL for each domain was between 44 and 56. This indicated that adolescents’ QoL in the Soreang subdistrict was moderate.

### 3.3. Association between Nutrient Intake and Quality of Life

According to bivariate analyses (Table 3), there was a significant weak positive correlation between parents’ education and the mothers’ occupations with the environmental domain (0.1 < *r* < 0.3, *p* < 0.05). On the other hand, there was a significant weak negative correlation between the fathers’ occupations with the psychological domain (−0.5 < *r* < −0.3, *p* < 0.05). Our results indicated that BMI had a significant weak negative correlation with the environmental domain (−0.5 < *r* < −0.3, *p* < 0.05). There was a significant weak positive correlation between energy, protein, carbohydrates, vitamin B6, magnesium, and zinc with physical health and the environmental domain (0.1 < *r* < 0.3, *p* < 0.05). Additionally, both energy and calcium had a significant weak positive correlation with the psychological domain (0.1 < *r* < 0.3, *p* < 0.05). A statistically significant weak positive association was observed between protein, carbohydrates, and zinc with social relationships (0.1 < *r* < 0.3, *p* < 0.05). A negative correlation between two variables indicates that one variable increase while the other decreases, and vice versa. This relationship may or may not represent causation between the two variables, but it describes an observable pattern. A negative correlation can be contrasted with a positive correlation, which occurs when two variables tend to move in tandem. Additionally, a weak correlation might be shown as a result of a limited sample.

### 3.4. Factors Associated with Quality of Life (QOL)

According to multiple linear regression models (Table 4), the strongest influencing factors for the QoL for each domain was (1) carbohydrates, vitamin C, and fat for physical health; (2) calcium for the psychological domain; (3) carbohydrates and vitamin C for social relationships; and (4) BMI and zinc for the environmental domain. However, there was a negative correlation between vitamin C and fat with physical health. Similarly, there was a negative correlation between vitamin C and social relationships. A similar finding showed a negative correlation between BMI and the environmental domain.

## 4. Discussion

### 4.1. Sociodemography, Nutrient Intake, and Quality of Life

Adolescence is a period of crucial development and nutritional vulnerability due to the increased requirements for energy and nutrients [26]. Our study revealed that during this critical time, participants’ nutrient intake was lower than the nutrient composition of Indonesia reference [18]. Therefore, a large number of Indonesian adolescents are undernourished [6]. This is in line with the study by Maehara et al. which reported that adolescents aged 15–18 years were more likely to be malnourished [27]. Girls in late adolescence are particularly prone to eating disorders; this vulnerability derives in part from profound anxieties over body image that are fueled by cultural and media stereotypes of feminine beauty [28]. Surprisingly, some participants had higher nutrient intakes than the reference, including higher energy, protein, carbohydrate, fat, vitamin A, vitamin B6, vitamin C, and zinc intakes. These conditions affect the nutritional status of adolescents, so that there are adolescents who experience deficiencies but there are also those who experience excess nutrition. Malnutrition in the form of both underweight and overweight adolescent girls is present in the Soreang subdistrict and rural areas of Indonesia. This is in line with the study in the Klaten and Lombok Barat districts showing that adolescents in Indonesia face both spectrums of malnutrition [27]. According to the National Basic Health Research Survey, the problem of both undernutrition and overnutrition in Indonesia requires critical consideration [29].

This study found that the majority of participants with an average weight consumed more macronutrients, specifically carbohydrates, than participants with abnormal weights. This similar with study conducted by Storey et al. that participants with an average weight consumed significantly more carbohydrates and fiber than overweight or obese participants [30]. Another reason that high carbohydrate intake high among average-weight participants is because rice is the leading food of Indonesia. There are times when many Indonesians consume only rice in their daily meals. Previous studies have indicated that a low carbohydrate intake improved the QoL, although this result was limited to two studies [7,31]. Another study has revealed that the physiological changes that occur when fatty acid is consumed may mainly influence the psychological QoL. Therefore, participants with the highest intake felt more tired, with social roles disabled due to emotional problems [32].

The study also highlighted that the majority of participants had lower micronutrient intakes than the standard. A similar finding was reported in Spanish female adolescents who had a low micronutrient intake [33,34]. Micronutrient deficits pose the highest nutritional risk [34]. Micronutrient deficiencies in adolescence can lead to impaired growth, delayed sexual maturation, and reproductive issues in adulthood [35,36]. Adequate calcium intake is essential for bone development and for the prevention of osteoporosis in adulthood [36,37]. One study revealed that school-aged adolescents had low iron intakes because of their large consumption of nonheme-rich food and food that inhibits iron absorption [36,38]. Essential micronutrients are also important for disease prevention during adulthood. Other examples of micronutrient deficiencies linked to disease and impairment are anemia (Fe, folic acid, Cu, Co, Mg, Zn, and vitamins B-12, B-6, and A,), birth defects (vitamin A and folate), cancer (vitamins E and C, folate, and Fe), central nervous system function (Fe, I, Se, and Zn), cognitive function (Fe, Zn, and vitamins B-1 and B-12), gene interactions (Fe, Zn, and vitamins B-6, C, and K), heart disease (vitamins E, C, B-6, and B-12, carotenoids, folic acid, and Fe), immune system development and host defense (Zn and vitamin E), and osteoporosis (Ca and vitamins D and K) [39].

Some studies state that adolescent intake of macro and micronutrients is associated with quality of life. This study showed the QoL scores (median, min–max) among adolescents in the domains of physical health (44, 25–81), psychological domain (56, 19–94), social relationships (56, 19–94), and environmental domain (56, 31–100). The results of this study were lower than the results seen in the study conducted by Purba et al. showing that adolescents’ QoL had the highest scores in overall QoL domains [15]. Other studies have indicated that adolescent girls showed the lowest general QoL [40]. This observation might reflect girls’ age-specific and body-related changes, especially after the onset of puberty. Our study also revealed that adolescents’ characteristics and nutrient intake correlated with some quality-of-life dimensions.

### 4.2. Association between Quality of Life and Nutrient Intake (Bivariate Analyses)

#### 4.2.1. Physical Health (Domain 1) and Nutrient Intake

There was a significant weak positive correlation between physical health and energy, protein, carbohydrate, vitamin B6, magnesium, and zinc. However, there was a negative correlation between vitamin C intake and physical health. There may be different possible explanations for this association. Vitamin C is very beneficial for the health of the body, but if consumed in excessive amounts will be not good for health. Accordingly, the optimal dose for vitamin C intake remains unanswered [41]. Vitamin C intake plays a major role in individuals’ health. Studies have shown that vitamin deficiencies may lead to poor QoL and cognitive decline [42]. Further research is needed to explore this relationship. In addition, a negative correlation was also found between fat and physical health, indicating that when the intake of fat increased, physical health decreased. This result is supported by Ruano et al., in which individuals with the highest fat intake perceived themselves as tired and worn out, and thus experienced social dysfunctions and role disabilities due to emotional problems [32]. This does not mean, however, that adolescents do not require an intake of fat. Rather, fat intake is required for the normal growth and development of adolescents.

#### 4.2.2. Psychological (Domain 2) and Nutrient Intake

This study showed a significant weak negative correlation between psychological domain and a fathers’ occupation. This may be because a fathers’ occupation is not the only one factor that impacts the QoL. Other factors influenced the QoL, such as physical activity [43], education levels and incomes [15], dietary intake, and nutritional status [33,44]. The results of this study are not consistent with the study that reported that besides nutrition intake, parents’ work also impacts adolescents’ QoL [45].

There was a significant weak positive correlation between the psychological domain and both energy and calcium. Our results revealed that calcium intake had a positive correlation with the psychological domain. Adolescents are in a stage of physical and psychological growth and development. Hormonal changes in adolescents sometimes cause fluctuations in psychological states, and stress (as well as hormones) is a major cause of these fluctuations. Other studies have determined that calcium is a major dietary need of adolescents as it is beneficial for bone growth, blood clotting, and muscle contraction. In nerve transmission, calcium releases neurotransmitters and permits ions’ flow into and out of nerve cells. Without sufficient calcium, nerve function fails, resulting in tetany. Finally, calcium helps regulate cellular metabolism by influencing the activities of various enzymes and hormonal responses, including coping with stress [46]. Our finding of the importance of calcium with the QoL’s psychological domain corroborated a study that showed that adolescents in India with low calcium levels were more prone to psychological disorders [47]. Several studies have shown that calcium plays a major role in health, especially bone health [48]. Aljaraedah et al. have shown that calcium intake is an essential requirement for development and physical growth and is critical in the formation of bone mass and in the reduction of the lifetime risk of osteoporosis [5]. The importance of calcium is thus evident in its influence on a person’s psychological condition, as shown in Table 4.

#### 4.2.3. Social Relationships (Domain 3) and Nutrient Intake

There was a significant weak positive correlation between social relationships and protein, carbohydrate, and zinc. This indicated that while both variables, nutrient intake and QoL, tend to increase in response to one another, the relationship is not very strong. This might be due to other influential QoL factors that must be considered. In spite of the relationship between diet and biological needs, the variety of ways in which diet relates to psychological, emotional, and social factors has led us to suspect that diet correlates with QoL dimensions [49]. Our study did not analyze processed food specifically, so the results of this study were limited to the kind of food that adolescents consume. Other than what we have shown in our results, there is no direct relationship between the intake of certain nutrients and the QoL.

Statistical analyses showed that carbohydrates and vitamin C were the most influential on social relationships. Carbohydrates are human’s main dietary source of energy. Previous studies have revealed that carbohydrate intake influences behavior and cognitive functions in adolescents [49]. Carbohydrates are components of macronutrients that provide calories and energy. Carbohydrate intake directly impacts body weight and BMI, which directly correlates with physical health [50,51]. A previous study revealed that obesity determined from BMI categories also correlated with QoL; this means carbohydrate intake indirectly correlated with QoL [52]. Surprisingly, Table 4 showed a negative correlation between vitamin C and social relationships. Actually, according to the bivariate analyses, there was no correlation, but the results showed a weak negative correlation in the multivariate analyses. This statistical analysis means that when vitamin C intake increased, the QoL decreased. This study only relies on vitamin C intake from the FFQ. Our findings agreed with the review by Lykkesfeldt indicating that there is no linear relationship between vitamin C intake and the level of vitamin C in the blood [41]. Moreover, the determination of vitamin C blood levels are only feasible through plasma analysis. Significantly, FFQ suffers from inaccuracy due to human recall error, inability to account for vitamin loss from storage and preparation of foods, and not accounting for polymorphisms possibly affecting vitamin C homeostasis [41,53,54,55].

#### 4.2.4. Environment (Domain 4) and Nutrient Intake

There was a significant weak positive correlation between the parents’ education and a mother’s occupation and the environmental domain. This was in accordance with a study by Purba et al., indicating that higher-educated, high-income respondents had the highest scores in overall QoL [15]. Furthermore, a negative correlation was observed between BMI and the environmental domain, indicating that when BMI increased, the environmental domain decreased. This is supported by studies conducted in other countries showing that the obese group had significantly lower scores in QoL than the normal-weight group [38,47,49]. Overweight or obese people are limited in their activity because of their additional weight and thus this limitation impacts their environmental domain. Furthermore, adolescents with higher BMIs often experience chronic health conditions that can impact their QoL. In addition, there was a significant weak positive correlation between the environmental domain and energy, protein, carbohydrate, vitamin B6, magnesium, and zinc. Variables that influenced the environmental domain were zinc and BMI. Based on previous research, zinc has shown a positive effect on psychological conditions [56]. Nevertheless, our study revealed a negative correlation between BMI and the environmental domain, which is depicted in the bivariate analysis.

### 4.3. Quality of Life and Nutrient Intake Multivariate Analyses

The strongest correlations were between (1) physical health with carbohydrates, vitamin C, and fat; (2) psychological domain with calcium; (3) social relationships with carbohydrates and vitamin C; and (4) environmental domain with BMI and zinc. Moreover, all macronutrient and micronutrient intakes were essential for adolescents’ growth and development. This result is supported by previous studies showing that nutritional factors are associated with the QoL of healthy populations [33,57,58].

## 5. Limitation and Recommendation

During the pandemic, it was challenging to include participants in this study, so the sample size was small, which was a limitation of our study. Our recommendation: To prevent malnutrition, it is essential to focus on adolescents’ nutritional status and intake. Health education is important, and it is necessary to consider the subject matter of nutrition in schools.

## 6. Conclusions

The study revealed the nutrient intake of adolescent girls was lower than the nutrient composition of the Indonesia reference. On the other hand, the QoL from the four domains (in median) was lower than 60. There was a significant positive correlation between the intake of some nutrients and adolescents’ QoL, despite the observation of some significant negative correlations. The strongest influence in the QoL for each domain was (1) carbohydrates, vitamin C, and fat for physical health; (2) calcium for the psychological domain; (3) carbohydrates and vitamin C for social relationships; and (4) BMI and zinc for the environmental domain. The findings of this study indicate that more attention should be focused on adolescents’ nutrient intake in order to improve their QoL.

## Figures and Tables

**Table 1:** Sociodemographic Characteristics.

Variables	Frequency (%)
*Age in Years*	
Median (Min–Max) = 17(15–19)	
*Father’s Education*	
Elementary	94(59.87)
Second and above	63(40.13)
*Mother’s Education*	
Elementary	105(66.88)
Secondary and above	52(33.12)
*Father’s Occupation*	
Farmer	5(3.18)
Trading	54(34.39)
Civil Servant	7(4.47)
Other ^1^	91(57.96)
*Mother’s Occupation*	
House Wife	136(86.6)
Trading	11(7.0)
Civil Servant	1(0.6)
Other ^1^	9(5.8)
*Living Condition*	
With the family	139(88.5)
At Dormitory	18(11.5)
*Number of Siblings*	
Only Child	4(2.5)
One	41(26.1)
Two or More	112(71.4)
*Body Mass Index (BMI)*	
Underweight	41(26.1)
Pre-obesity	17(10.9)
Obesity-2	1(0.6)
Obesity-1	4(2.5)
Normal	94(59.9)

Other ^1^ daily laborer, a private employee, has died, unemployed.

**Table 2:** Nutrient Intake and Quality of Life.

Variables	Minimum	Median	Maximum	Recommendation *
***Nutrient***				
Energy (kcal)	195.7	908.25	2407.39	2125–2250
Protein (g)	3.82	24.16	115.08	56–69
Carbohydrate (g)	18.39	128.89	374.83	292–309
Fat (g)	2.67	21.89	100.43	71–75
Vit A (mg)	4.46	77.1	686.34	600
Vit E (mg)	0.01	1.4	11	15
Vit B1 (mg)	0.05	0.19	0.85	1.1
Vit B2 (mg)	0.06	0.29	1.45	1.0–1.1
Vit B6 (mg)	0.09	0.45	3.05	1.2–1.3
Folic Acid (mg)	7.4	35.13	224.4	400
Vit C (mg)	0	12.6	323.46	65–75
Calcium (mg)	13.96	197.46	2921	1000–1200
Magnesium (mg)	21.84	93.72	355.5	220–230
Iron (mg)	0.5	2.64	12.15	26
Zinc (mg)	0.42	2.09	14.5	8–9
***Quality of Life***				
Physical Health	25	44	81	
Psychological Domain	19	56	94	
Social Relationship	19	56	94	
Environmental Domain	31	56	100	

* The nutrient composition of Indonesia reference [18].

**Table 3:** Correlation of sociodemographic variables, nutritional status, nutrient intake, and their association with the QoL among adolescent girls.

Variables	Physical Health		Psychological Domain		Social Relationships		Environment Domain	
	*r*	*p*	*r*	*p*	*r*	*p*	*r*	*p*
*Father’s Education*	0.057	0.547	−0.075	0.362	−0.005	0.952	0.228	0.008
Elementary								
Secondary and above								
*Mother’s Education*	−0.042	0.632	0.089	0.328	0.089	0.328	0.243	0.003
Elementary								
Secondary and above								
*Father’s Occupation*	0.03	0.738	−0.177	0.042	−0.003	0.973	−0.040	0.635
Farmer								
Trading								
Civil Servant								
Other^1^								
*Mother’s Occupation*	0.11	0.481	−0.014	0.925	0.249	0.108	0.367	0.022
Housewife								
Trading								
Civil Servant								
Other								
*Living Condition*	−0.162	0.221	−0.019	0.876	0.087	0.433	0.049	0.67
With the family								
At Dormitory								
*Number of Siblings*	0.073	0.551	0.193	0.091	0.115	0.317	0.047	0.666
Only Child								
One								
Two or More								
*Body Mass Index*	−0.102	0.318	0.001	0.99	−0.070	0.468	−0.206	0.027
Underweight								
Pre-obesity								
Obesity-2								
Obesity-1								
Normal								
*Nutrient*								
Energy (kcal)	0.226	≤0.001	0.126	0.047	0.124	0.056	0.147	0.012
Protein (g)	0.207	≤0.001	0.119	0.066	0.135	0.043	0.133	0.026
Carbohydrate (g)	0.255	≤0.001	0.11	0.08	0.139	0.026	0.17	0.003
Fat (g)	0.055	0.416	0.132	0.052	0.047	0.49	0.031	0.615
Vit A (mg)	0.03	0.68	0.033	0.626	0.024	0.716	0.071	0.266
Vit E eq (mg)	−0.072	0.3	−0.005	0.932	−0.055	0.363	−0.58	0.378
Vit B1 (mg)	0.084	0.23	0.001	0.986	0.023	0.719	0.066	0.284
Vit B2 (mg)	0.098	0.167	−0.015	0.805	0.072	0.229	0.11	0.082
Vit B6 (mg)	0.146	0.037	0.01	0.872	0.069	0.327	0.135	0.034
Folic Acid	0.065	0.363	0.032	0.626	0.04	0.536	0.088	0.156
Vit C (mg)	−0.064	0.317	−0.023	0.709	−0.078	0.173	−0.043	0.444
Calcium (mg)	0.067	0.314	0.186	0.002	0.066	0.339	0.046	0.469
Magnesium (mg)	0.175	0.006	0.046	0.49	0.075	0.25	0.15	0.017
Iron (mg)	0.093	0.163	0.021	0.752	0.035	0.58	0.1	0.12
Zinc (mg)	0.27	≤0.001	0.061	0.351	0.141	0.03	0.226	≤0.001

*p* value significant at <0.05 based on the chi-square test. Other ^1^ daily laborer, a private employee, has died, unemployed.

**Table 4:** Multiple linear regression models to identify factors associated with QoL among adolescent girls in the Jatinangor District, a rural area of Indonesia.

Dependent Variable	Coefficients				
	Step	Model	B	β	*p*
Physical Health ^a^	3	(Constant)	42.895		≤0.001
		Carbohydrate (g)	0.070	0.517	≤0.001
		Vit C (mg)	−0.044	−0.235	0.002
		Fat (g)	−0.099	−0.196	0.036
Model Summary R = 0.433; R^2^ = 0.188; adj R^2^ = 0.172; SEE^g^ = 8.976 ANOVA: F = 11.797; *p* ≤ 0.001					
Psychological Domain ^b^	1	(Constant)	55.770		≤0.001
		Calcium (mg)	0.005	0.203	0.011
Model Summary R = 0.203; R^2^ = 0.041; adj R^2^ = 0.035; SEE^g^ = 12.596 ANOVA: F = 6.683; *p* > 0.001					
Social Relationships ^c^	2	(Constant)	54.487	-	≤0.001
		Carbohydrate (g)	0.049	0.256	0.002
		Vitamin C (mg)	−0.055	−0.211	0.009
Model Summary R = 0.288; R^2^ = 0.083; adj R^2^ *=* 0.071; SEE = 13.265 ANOVA: F = 6.647; *p* > 0.011					
Environment Domain ^d^	2	(Constant)	117.351	-	≤0.001
		Body Mass Index	−0.979	−0.138	0.060
		Zinc (mg)	2.125	0.264	≤0.001
Model Summary R = 0.346; R^2^ = 0.120; adj R^2^ *=* 0.109; SEE = 12.027 ANOVA: F = 10.496; *p* ≤ 0.001					

^a^. Excluded variables: living condition, vitamin B2(mg), carbohydrates (g), zinc (mg), vitamin B1 (mg), energy (kcal). ^b^. Excluded variables: father’s occupation. ^c^. Excluded variables: energy (kcal), vitamin B2 (mg), protein (g), zinc (mg), mother’s occupation. ^d^. Excluded variables: magnesium (mg), father’s education, total folic acid (µg), mother’s occupation, vitamin B6 (mg), iron (mg), protein (g), vitamin B2 (mg), energy (kcal), carbohydrates (g); SEE^g^ = standard error of estimate.

## Data Availability

Not applicable.
